# Late Effects: Focus on Adolescent and Young Adult Cancer Survivors

**DOI:** 10.6004/jadpro.2016.7.1.1

**Published:** 2016-01-01

**Authors:** Pamela Hallquist Viale

One of the most rewarding things about having worked in oncology for over 30 years has been celebrating the improvements in cancer survival. However, these improvements and higher cure rates come with their own complications. These patients require complex survivorship care, adding to the financial burden of cancer care itself. For younger patients, the challenges of survivorship and the long-term effects of therapy can be especially challenging.

**Survival Rates in Children**

Although the incidence of childhood cancers increased from 1975 through 2006 (according to Surveillance, Epidemiology, and End Results Program [SEER] data), the childhood cancer mortality rates decreased by more than 50% between those same years (Smith et al., 2010). In the 1-to-14–year age group, 5-year survival rates have increased to 80.6% in 1999–2002, from approximately 60% measured in 1975–1978 (Smith et al., 2010).

For surviving children and young adults with cancer, survivorship care becomes an integral part of their lives, requiring monitoring for a myriad of physical complications that can affect their entire lives. And the psychological and neurocognitive function in young patients can also be a considerable concern after cancer therapy.

**Late Effects of Therapy**

These psychological and neurologic side effects can be varied and include reductions in IQ scores and cognitive functioning. Changes can be long-standing and were documented in 28.6% to 58.9% of children treated for acute lymphoblastic leukemia in one published study (Krull et al., 2013). Most of the participants were aged 33 years, with 26 years having passed since their original diagnosis; the survivors experienced persistent and measurable neurocognitive changes (Krull et al., 2013). Changes in intelligence, academic performance, and memory were consistent with previous reports; in this study, the risk for impairment decreased gradually with the increased age of the child at treatment (Krull et al., 2013).

The positive news that 400,000 survivors of childhood and early young adult cancer are alive today is an example of the results of better therapies and innovative agents to combat cancer. But this population of patients is also at risk for late effects from their therapies, including morbidities creating both physical and neurologic problems. Much of the previous research has been with younger children; however, psychological and neurocognitive functioning in adolescent and early young adults was reported in a recently published study (Prasad et al., 2015), and the results were particularly striking.

**Neurocognitive Dysfunction in Adolescents and Young Adults**

The early years of the adolescent and young adult period are usually referred to as the ages between 11 and 21 years, a time where neurocognitive function is developed and behavior patterns are established (Prasad et al., 2015). The researchers studied 6,192 survivors of cancer and 390 siblings who completed the Brief Symptom Inventory-18 and a neurocognitive questionnaire. The aim of the research study was to look at self-reported psychological symptoms and complaints of neurocognitive dysfunction during those critical years as well as to identify important risk factors for this population. The authors of the study also examined treatment and demographic predictors as well as associations with employment, education, and ability to live independently (Prasad et al., 2015).

**Study Results**

This study is the first to report on long-term neurocognitive functional changes after treatment of cancer during adolescent and young adult years. And although it has long been known that childhood cancer survivors suffer impaired neurocognitive function, this study pointed out that the adolescent and young adult survivors demonstrate increased rates of emotional distress and neurocognitive dysfunction when compared with their sibling study participants. These survivors were less likely to go on to higher education, had decreased rates of full-time employment, and were less likely to be living independently—all important markers of adulthood (Prasad et al., 2015).

**Implications for Advanced Practitioners**

Those advanced practitioners caring for adolescent and young adult patients with cancer are seeing larger numbers of these patients cured, a fact we can celebrate. As those patients become survivors and age into the next decade, all of the tasks and milestones necessary for successful integration into adulthood become critical. As advanced practitioners follow these patients and provide essential survivorship care, we must also recognize the risk for impaired neurocognitive functioning and support this young adult population.

The National Comprehensive Cancer Network (NCCN) has published guidelines that are unique to this group of patients, identifying late effects that although uncommon may include central nervous system tumors and changes in executive function, attention span, memory, and the ability to process (NCCN, 2015). The NCCN also recommends that in some patients, formal neuropsychological evaluation should be performed (NCCN, 2015). The NCCN offers a patient-centered version of the guideline as well.

The ability to cure more young patients is a positive and welcome improvement in cancer care; the need to monitor these patients for neurocognitive changes following those successful treatments is a necessary survivorship mandate.
